# Orthogonal *in vivo* and *in vitro* membrane engineering enables human-like lipid remodeling of bacterial magnetosomes for functional TrkA display

**DOI:** 10.1128/aem.01710-25

**Published:** 2025-11-05

**Authors:** Ryoto Tomoe, Shunya Waki, Keita Morimoto, Takaho Ogaki, Tsuyoshi Tanaka, Tomoko Yoshino

**Affiliations:** 1 Division of Biotechnology and Life Science, Institute of Engineering, Tokyo University of Agriculture and Technology 98386 , Koganei, Tokyo, Japan; University of Illinois Urbana-Champaign, Urbana, Illinois, USA

**Keywords:** tropomyosin receptor kinase A, magnetosome, magnetotactic bacterium, cholesterol, lipid membrane

## Abstract

The magnetotactic bacterium *Magnetospirillum magneticum* produces biogenic magnetic nanoparticles called magnetosomes, each comprising a magnetite core surrounded by a lipid bilayer. By expressing the human neurotrophin receptor TrkA, which is implicated in Alzheimer’s disease, cancer, and other pathologies, on magnetosomes, we created a functional human transmembrane receptor platform that enables magnetic recovery and ligand screening. However, TrkA has limited activity within the native bacterial membrane, likely due to the absence of key eukaryotic lipids such as phosphatidylcholine (PC) and cholesterol (Chol). To overcome this limitation, we combined *in vivo* and *in vitro* membrane engineering to remodel the magnetosome envelope and closely mimic the human cell membrane. Specifically, PC was biosynthesized *in vivo* by co-expressing phosphatidylcholine synthase, and purified magnetosomes were subsequently enriched *in vitro* with Chol using methyl-β-cyclodextrin. This dual-lipid strategy significantly enhanced TrkA-ligand-binding and autophosphorylation. These findings show that orthogonal membrane engineering can precisely tailor the lipid composition of bacterial magnetosomes to recreate mammalian-like bilayers, rescuing the function of complex human receptors in a microbial environment. This approach expands the metabolic and synthetic biology toolkit for producing functional membrane proteins on scalable magnetic supports and establishes a cost-effective platform for the discovery of TrkA and other membrane receptors.

**IMPORTANCE:**

Membrane receptors drive essential signaling in health and disease; however, they are difficult to study and screen because most platforms fail to reproduce human-like membranes at scale. Magnetosomes from the bacterium *Magnetospirillum magneticum* AMB-1 offer a simple alternative: lipid-bounded, magnetic nanoparticles that can be purified in one step. This work establishes human-like remodeling of magnetosome membranes by combining *in vivo* phosphatidylcholine synthesis with the first *in vitro* cholesterol loading of these particles. Displaying human tropomyosin receptor kinase A on the remodeled membranes preserved and enhanced the receptor function without complex purification or reconstitution. Because magnetosomes can be produced inexpensively and recovered magnetically, this approach enables practical, high-throughput assays for ligand discovery and inhibitor testing. The strategy is broadly applicable to other human membrane proteins, linking microbial biotechnology with human membrane biology to accelerate translational research.

## INTRODUCTION

Tropomyosin receptor kinase A (TrkA) is widely expressed in the peripheral and central nervous systems, immune tissues, and various peripheral organs. It regulates essential aspects of neuronal development and function, including differentiation, growth, and synaptic plasticity (1). TrkA is activated by the binding of nerve growth factor (NGF), which triggers receptor dimerization and autophosphorylation, initiating downstream signaling (2). Dysregulated TrkA-NGF signaling is implicated in diseases such as Alzheimer’s disease, cancer, and neuropathic pain (3), highlighting TrkA as a key target for drug discovery (4, 5). As a transmembrane receptor, TrkA requires a lipid bilayer environment to maintain its structural stability and function. Artificial systems, such as liposomes and nanodiscs, have been used to reconstitute membrane proteins for high-throughput screening; however, challenges remain in terms of procedural complexity, limited scalability, high cost, and suboptimal biomimicry (6, 7).

We propose an alternative platform based on magnetosomes, nanoscale magnetic particles surrounded by a lipid membrane and biosynthesized by *Magnetospirillum magneticum*. Target membrane proteins can be expressed in the magnetosome membrane through protein engineering (8). Using this system, we have successfully displayed TrkA and other human transmembrane receptors, including G protein-coupled receptors (GPCRs), on magnetosomes, enabling the construction of membrane protein libraries for functional screening (9–11). Membrane protein-magnetosome complexes produced by *M. magneticum* can be isolated by simple cell disruption followed by magnetic separation, eliminating chromatographic purification and membrane reconstitution. Low cultivation costs further enhance practicality, offering several advantages over liposomes and nanodisks. However, the target membrane receptors displayed on magnetosomes exhibit reduced ligand-binding affinity. We previously developed a TrkA-magnetosome complex; however, its binding affinity for NGF was approximately two orders of magnitude lower than that observed in native mammalian membranes (9). This reduction is likely due to the phospholipid-dependent microenvironment of the membrane, which plays a critical role in maintaining the structural integrity and function of the embedded transmembrane proteins (12).

It is well established that bacterial and eukaryotic membranes differ significantly in lipid composition. In *M. magneticum* and other bacteria, membranes primarily comprise phosphatidylethanolamine (PE) and phosphatidylglycerol (PG), whereas they lack phosphatidylcholine (PC) and cholesterol (Chol), two major components of eukaryotic membranes. Besides PC and Chol, eukaryotic membranes are enriched in phosphatidylserine (PS), phosphatidylinositol (PI), and sphingomyelin (SM) (13, 14). We recently introduced PC into *M. magneticum* using a genetic engineering approach involving phosphatidylcholine synthase (PCS) (15, 16). The incorporation of PC into magnetosome membranes improved ligand binding to the thyroid-stimulating hormone receptor (TSHR), a representative GPCR. Based on these results, we focused on cholesterol. Cholesterol modulates lipid bilayer properties, including fluidity, thickness, compressibility, water permeability, and curvature, influencing membrane protein function through multiple mechanisms (17). Recent studies have shown that cholesterol affects the structure and function of GPCRs (18, 19), transporters, and ion channels (20, 21). Therefore, further mimicking the composition of mammalian membranes by incorporating cholesterol into magnetosomes may enhance the functional performance of TrkA, which is displayed on the magnetosome surface.

In this study, we aimed to mimic the native membrane environment of magnetosomes by incorporating cholesterol *in vitro* using methyl-β-cyclodextrin (MβCD), which enables the efficient delivery of hydrophobic molecules into lipid bilayers (22). We evaluated whether the dual incorporation of PC and cholesterol enhanced TrkA functionality in magnetosomes. This approach combines synthetic membrane remodeling with microbial membrane protein display, providing a scalable and genetically tunable platform for studying transmembrane receptor function under near-physiological conditions. Our findings advance bacterial membrane engineering strategies for membrane protein applications and support the development of cost-effective systems for ligand screening and receptor-targeted drug discovery applications.

## MATERIALS AND METHODS

### Bacterial strains and growth conditions

*Escherichia coli* TOP10 (Thermo Fisher Scientific, Waltham, MA, USA) served as the cloning host and was cultured in lysogeny broth (LB) containing 50 µg/mL ampicillin at 37°C. *M. magneticum* AMB-1 (ATCC 700264) was grown microaerobically in magnetospirillum growth medium (MSGM) at 28°C under an argon atmosphere, as described previously (23). AMB-1 transformants were selected on MSGM supplemented with 5 µg/mL of ampicillin. The expression of trkA and phosphatidylcholine synthase was induced in the mid-log phase with anhydrotetracycline (ATc, Kanto Chemical, Tokyo, Japan) (final concentration 100 ng/mL); 2 mM choline (Wako, Tokyo, Japan) was added simultaneously to the PCS-expressing cultures.

### Preparation of TrkA magnetosomes and TrkA-PCS magnetosomes

Following our previous protocol (15), we constructed two plasmids: pUMtOR13-TrkA, which expresses TrkA, and pUMtOR13-TrkA-PCS, which co-expressed TrkA and PCS. Each plasmid was introduced into *M. magneticum* AMB-1 by electroporation, and transformants were selected on MSGM supplemented with ampicillin (5 µg/mL). *M. magneticum* cells were collected by centrifugation at 8,000 × *g* for 10 min at 4°C, resuspended in 10 mM 4-(2-hydroxyethyl)-L-piperazineethanesulfonic acid (HEPES) buffer (pH 7.4), and disrupted by three times using a French press at 1,500 kg/cm^2^ (Ohtake Works Co. Ltd., Tokyo, Japan). The magnetosomes were collected from the disrupted cells using a columnar neodymium-iron-boron (Nd-Fe-B) magnet and washed by repeating the washing process (resuspension of magnetosomes in HEPES buffer, sonication, and magnetic separation) 10 times. The washed magnetosomes were suspended in HEPES buffer. The concentration of magnetosomes in the suspension was determined by measuring the optical density at 660 nm using a spectrophotometer (UV-2200, Shimadzu Co., Kyoto, Japan).

### Extraction of polar lipids

Lyophilized cells (25 mg) or magnetosomes (1 mg) were suspended in 1 mL of methanol: 0.3% NaCl aqueous solution (10:1, vol/vol) and heated at 100°C for 5 min, followed by cooling to 37°C for 5 min. Subsequently, 1.15 mL of chloroform:methanol:water (9:10:3, vol/vol/vol) was added and incubated for 1 h at room temperature (26°C). The samples containing lyophilized cells were centrifuged at 1,000 × *g* for 10 min, and the resulting supernatant was collected. Samples containing magnetosomes were magnetically separated using an Nd-B magnet to remove the magnetite cores, and the supernatant was collected. The separated cell pellets or magnetite cores were subjected to a second round of extraction by re-suspending them in 375 µL of chloroform:methanol:water (5:10:4, vol/vol/vol). The suspension was then rotated for 30 min. Next, 625 µL of chloroform and the same volume of 0.3% NaCl aqueous solution were added, followed by centrifugation at 1,000 × *g* for 10 min. The resulting organic phase was collected and combined with the first-round extracts. After evaporating the organic solvent with Ar gas, the dried extracts were dissolved in a chloroform:methanol mixture (2:1, vol/vol) to a final concentration of 20 mg/mL.

### Thin-layer chromatography

The lipids extracted from the lyophilized cells or magnetosomes (40 µg and 120 µg of lipids, respectively) were spotted on silica gel high-performance thin-layer chromatography (TLC) plates (Glass HPTLC Silica gel 60 plates, Merck KGaA, Darmstadt, Germany), which were preheated at 120°C for 30 min before use. 1,2-dipalmitoyl-sn-glycero-3-phosphoethanolamine, 1,2-dioleoyl-sn-glycero-3-phospho-rac-(1-glycerol) sodium salt, and 1,2-dipalmitoyl-sn-glycero-3-phosphocholine were used as commercially available standards for PE, PG, and PC, respectively (Tokyo Chemical Industry Co., Ltd., Tokyo, Japan). The same weights of PE, PG, and PC were pre-mixed and spotted on the plates. Lipids were separated using chloroform:methanol:acetic acid (65:25:10, vol/vol/vol) as the developing solvent. After separation, the solvents were evaporated from the plates, and the spots of the separated lipids were visualized by exposing the plates to iodine vapor. Spot areas were measured as described in our previous report (15).

### *In vitro* cholesterol loading

**Fig 1:**
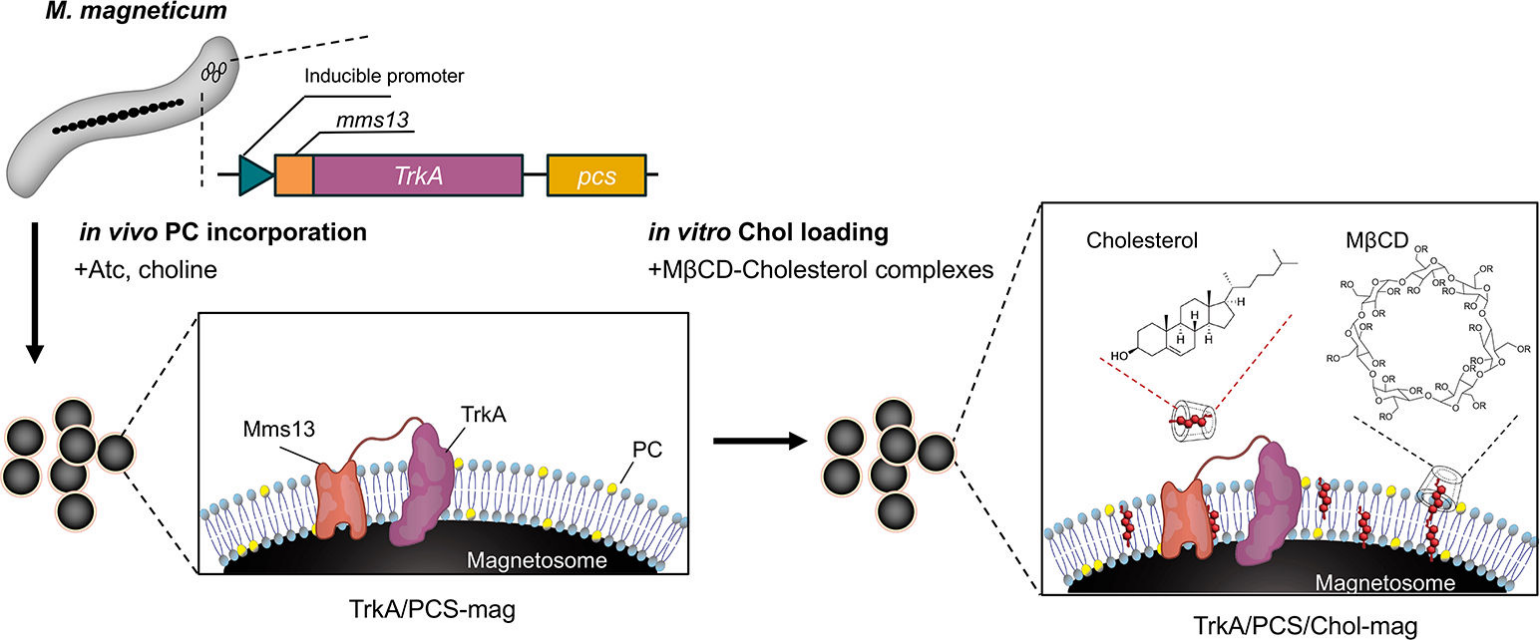
Schematic illustration of *in vivo* PC incorporation and *in vitro* cholesterol loading. Phosphatidylcholine synthase (PCS) was expressed under an anhydrotetracycline (ATc)–inducible promoter; in the presence of ATc and exogenous choline, PCS converts endogenous precursors into phosphatidylcholine (PC), which is inserted into the magnetosome membrane. The receptor tyrosine kinase TrkA was simultaneously displayed on the magnetosome surface via fusion to the native anchor protein Mms13. For *in vitro* cholesterol loading, methyl-β-cyclodextrin (MβCD)—a cyclic oligosaccharide that forms inclusion complexes with sterols at defined molar ratios—delivers cholesterol directly into the remodeled PC-containing lipid bilayer.

Cholesterol (C8667-5G, Sigma-Aldrich, St. Louis, MO, USA) was incorporated into the magnetosome membrane using a methyl-beta-cyclodextrin (MβCD; 320-84252, Wako)-cholesterol complex (Fig. 1). The complex was prepared by dissolving MβCD and cholesterol in a ratio of 1:10 (mol/mol) in 50 mM 2-amino-2-hydroxymethyl-propane-1,3-diol (Tris) buffer (pH 7.4) by constant shaking at room temperature (26°C) for 30 min. Solutions (1:10 mM cholesterol:MβCD) of this complex were prepared before each experiment. Cholesterol incorporation was performed at a concentration of 1 mg-Mag/mL by incubating each magnetosome with the cholesterol-MβCD complex solution for 30, 60, or 120 min at room temperature (26°C) under constant shaking (100 rpm) (NR-80, TATITEC Co., LTD., Saitama, Japan) or sonication (W-170ST, HONDA ELECTRONICS Co., LTD., Tokyo, Japan). The magnetosomes were then washed three times with Tris buffer and stored at 4°C until use. The concentration of the magnetosome was determined using the same methods as above, and the cholesterol content of the magnetosome membrane was estimated using the Amplex Red cholesterol assay kit (Thermo Fisher Scientific).

### Western blotting

Membrane proteins were extracted from 2.0 mg of magnetosomes from wild-type and transformant cells. The membrane proteins were mixed with an equal volume of 3× sodium dodecyl sulfate (SDS) sample buffer (125 mM Tris-HCl [pH 6.8], 10% 2-mercaptoethanol, 4% SDS, 10% sucrose, and 0.004% bromophenol blue) and denatured at 99°C for 15 min. The samples were separated on a 12.5% (wt/vol) gel using SDS-polyacrylamide gel electrophoresis (PAGE) and then transferred to a polyvinylidene difluoride membrane. FLAG tags were detected using 4 mL of 0.67 µg/mL mouse anti-FLAG (M2) monoclonal antibody (Sigma-Aldrich) and 4 µL of 0.025 µg/mL of HRP-conjugated goat anti-mouse IgG antibody (Proteintech, Rosemont, IL, USA) in Tris-buffered saline containing 0.1% (vol/vol) Tween-20 (TBST, 50 mM Tris, 150 mM NaCl, pH 7.6). Western Blot Ultra Sensitive HRP Substrate (Takara Bio Inc., Shiga, Japan) was used as the HRP substrate for protein visualization. Image analysis was performed using Image Studio, version 5.2. software (LI-CORbio, Lincoln, NE, USA).

### β-NGF-binding assay

Magnetosomes (50 µg) were incubated in blocking buffer (0.1% Tween 20 and 1% bovine serum albumin in TBS) for 1 h at 4°C to reduce nonspecific binding. The magnetosomes were then reacted with recombinant human β-NGF (50–800 nM; Pepro Tech, NJ, USA) and incubated for 1 h at 4°C in 100 µL of TBST after pulsed sonication. After three washes, rabbit anti-NGF antibody (5 µg/mL; Abcam, Cambridge, MA, USA) was added to the magnetosomes and incubated for 30 min at 4°C after pulsed sonication. After three washes, alkaline phosphatase-conjugated anti-rabbit IgG (whole molecule) antibody (5 µg/mL; Sigma-Aldrich) was added to the magnetosomes and incubated for 30 min at 4°C after pulsed sonication. After three washes, the magnetosomes were resuspended in 50 µL TBS, followed by the addition of 50 µL Lumi-Phos Plus. The luminescence intensity was measured as described above. Kd (dissociation constant) values were determined by performing a Scatchard analysis of the NGF-binding assay data.

### Autophosphorylation assay

Magnetosomes (50 µg) were incubated in blocking buffer for 1 h at 4°C. The magnetosomes were then reacted with recombinant human β-NGF (50–800 nM) and incubated for 1 h at 4°C in 100 µL of TBST after pulsed sonication. After three washes, the magnetosomes were resuspended and incubated in kinase buffer (20 mM HEPES, 4 mM MnCl_2_, 4 mM MgCl_2_, and 62.5 µM ATP) for 1 h at room temperature after pulsed sonication. After three washes, rabbit anti-phospho-TrkA (Y490) IgG antibody (5 µg/mL; Abcam) was added to the magnetosomes and incubated for 30 min at 4°C after pulsed sonication. After three washes, alkaline phosphatase-conjugated anti-rabbit IgG (whole molecule) antibody (5 µg/mL; Sigma-Aldrich) was added to the magnetosomes and incubated for 30 min at 4°C after pulsed sonication. After three washes, the magnetosomes were resuspended in 50 µL TBS, followed by the addition of 50 µL Lumi-Phos Plus. The luminescence intensity was measured as described above.

### Kinase activity assay

The kinase activity of TrkA expressed on magnetosomes was estimated using the Universal Tyrosine Kinase Assay Kit (Takara Bio Inc., Shiga, Japan). Magnetosomes (100 µg) were reacted with recombinant human β-NGF (0 or 500 nM in TBS) and incubated for 1 h at 4°C in 100 µL TBS. After three washes with TBST, the magnetosomes were resuspended in kinase reaction buffer and removed from the synthetic peptide-bound microplate. ATP-2-Na solution was added to each well and then incubated for 30 min at 37°C. After washing four times, the magnetosomes were incubated with blocking buffer for 30 min at 37°C. After discarding the supernatant, 50 µL/well of POD-conjugated anti-phosphorylated tyrosine antibody (Merk Millipore, Burlington, Massachusetts, USA) was added and incubated for 30 min at 37°C. The supernatant was discarded, washed four times with PBST, and incubated with 100 µL/well of TMBZ for 15 min at 37°C. The reaction was stopped by adding 100 µL/well of 1N sulfuric acid, and the absorbance was measured at 450 nm using a microplate reader.

## RESULTS AND DISCUSSION

### Cholesterol incorporation into magnetosome membranes using MβCD

**Fig 2:**
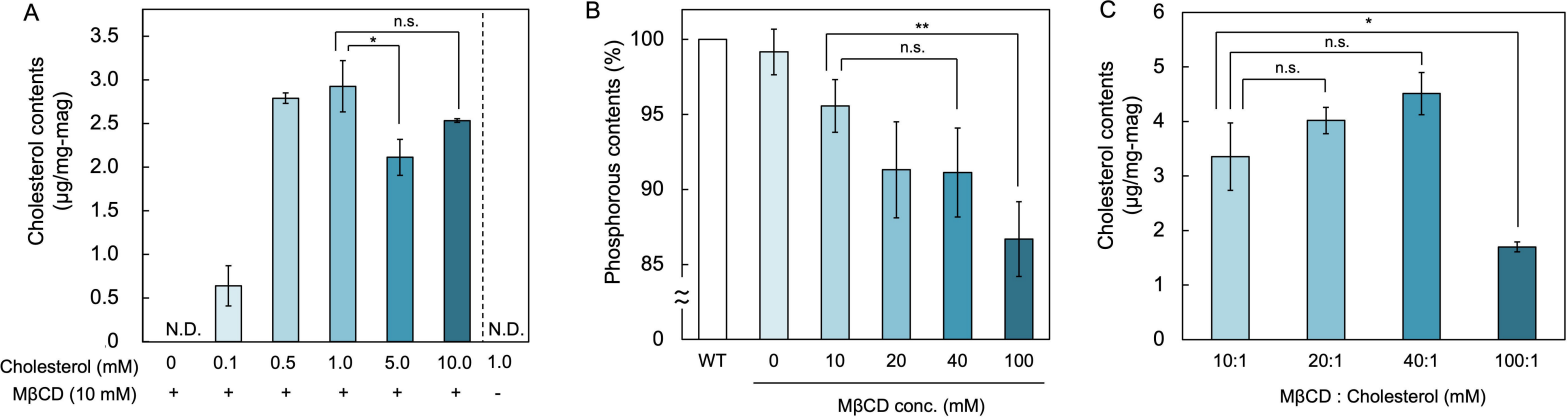
Cholesterol loading into magnetosome membranes using MβCD-cholesterol complexes. (**A**) Cholesterol incorporated per milligram of magnetosomes at increasing cholesterol inputs (cholesterol:MβCD, mM/mM) for 30 min at room temperature under shaking. Maximum loading (2.9 µg/mg-mags) was achieved at 1.0 mM cholesterol; higher concentrations did not increase the incorporation. (**B**) Residual membrane phospholipids after treatment with “empty” MβCD. Phosphate analysis showed a concentration-dependent loss of phospholipids, which fell below 95% of the untreated control at MβCD ≥ 20 mM. (**C**) Cholesterol incorporation after prior depletion of phospholipids. No significant differences were observed among the 10:1 to 40:1 cholesterol:MβCD ratios, whereas the 100:1 condition yielded significantly lower loading. Data are shown as mean ± SD (*n* = 3 biological replicates). Statistical significance was evaluated using a two-tailed Welch’s *t*-test; *P < 0.05; **P < 0.01; n.s., no significant difference. N.D., not detected.

Prokaryotes lack the enzymes required for cholesterol biosynthesis and cannot synthesize cholesterol. Moreover, cholesterol synthesis in eukaryotes is a complex multistep pathway, and it has been presumed that producing cholesterol in *M. magneticum* through heterologous expression would be difficult. Therefore, as an alternative approach, we attempted to incorporate cholesterol into magnetosome membranes *in vitro* using methyl-β-cyclodextrin (MβCD), which is commonly used to deliver cholesterol to lipid bilayers. Cholesterol-MβCD complexes were prepared at varying molar ratios and incubated with 1 mg of purified magnetosomes at room temperature (26°C) with shaking for 30 min. Cholesterol quantification revealed successful incorporation into the magnetosome membrane, with the highest level observed at a cholesterol:MβCD ratio of 1.0:10 (mM/mM), reaching 2.9 µg of cholesterol per mg of magnetosome (Fig. 2A). MβCD is a cyclic oligosaccharide composed of α−1,4-linked glucopyranose units that form a hydrophilic exterior and a hydrophobic cavity capable of encapsulating small hydrophobic molecules, such as cholesterol (22).

This property allows MβCD to serve as a vehicle for cholesterol delivery, as well as for cholesterol depletion from lipid membranes. However, when used in excess, MβCD can extract endogenous phospholipids, potentially disrupting the structural integrity of the lipid bilayer (24). To evaluate this risk, we incubated magnetosomes with empty MβCD alone and measured the membrane phosphate content. A concentration-dependent decrease in phosphate was observed, with over 8% depletion at MβCD concentrations above 20 mM (Fig. 2B). At 100 mM MβCD, approximately 13% of the phospholipids were removed. When cholesterol was added under these phosphate-depleted conditions, no significant improvement in cholesterol incorporation was observed compared to the 10:1 ratio (Fig. 2C). Cholesterol incorporation was reduced under the 100:1 condition, indicating that phosphate depletion does not facilitate cholesterol incorporation. These results indicate that cholesterol is most efficiently incorporated into the magnetosome membrane at a cholesterol:MβCD molar ratio of 1.0:10 without prior phospholipid depletion.

**Fig 3:**
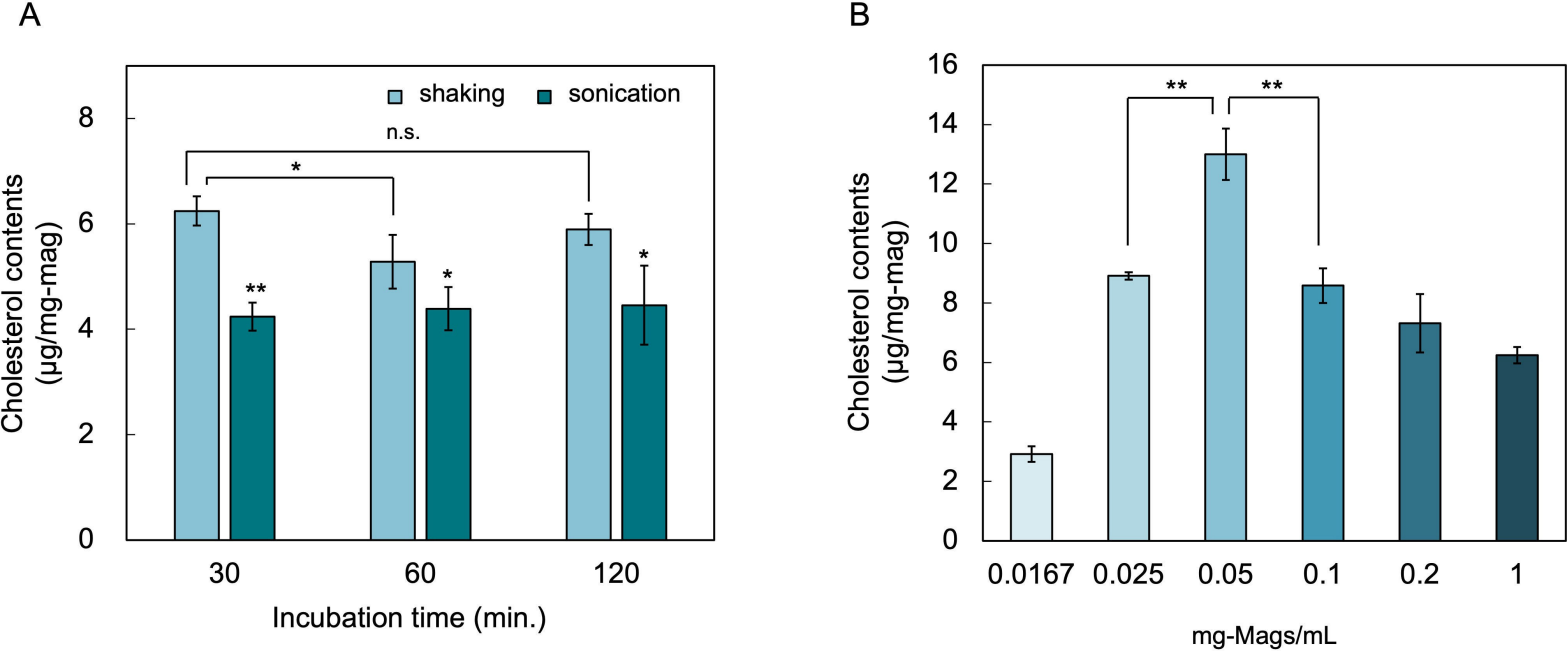
Optimization of experimental parameters for cholesterol loading into magnetosome membranes. (**A**) Magnetosomes were incubated with a pre-formed MβCD-cholesterol complex and subsequently mixed either by shaking or sonication for the indicated time points (30, 60, and 120 min). Sonication consistently reduced cholesterol incorporation compared to shake mixing. (**B**) Effect of magnetosome particle concentration on cholesterol loading. Maximum loading was obtained at 0.05 mg-mag/mL; both higher and lower particle concentrations yielded lower incorporation. Data are shown as mean ± SD (*n* = 3 biological replicates). Statistical significance was evaluated using a two-tailed Welch’s *t*-test; *P < 0.05; **P < 0.01; n.s., no significant difference.

To further enhance cholesterol incorporation into the magnetosome membranes, we evaluated the effects of sonication, reaction time, and magnetosome particle concentration. Magnetosomes are naturally dispersed because of their lipid bilayer coating, but usually aggregate upon prolonged standing. Sonication effectively redistributes the aggregated particles. However, a comparison of shaking and sonication at varying reaction time points revealed that sonication reduced the cholesterol incorporation efficiency (Fig. 3A). The reaction time had no measurable effect on cholesterol loading. Previous studies on cholesterol delivery using MβCD-Chol complexes have shown that the time required to reach equilibrium varies depending on the membrane system. For instance, cholesterol incorporation into CHO cell membranes increases over several hours (25), whereas incorporation into rod outer segment membranes reaches equilibrium within 2 h (26). In contrast, the magnetosomes reached apparent equilibrium within 30 min, likely due to their nanoscale size and high surface-to-volume ratio.

Based on these results, a 1.0:10 (mM/mM) cholesterol:MβCD ratio with 30 min of shaking was adopted as the standard loading protocol. We then investigated the effect of the magnetosome particle concentration. Cholesterol incorporation increased as the magnetosome concentration decreased, reaching a maximum of 13.0 µg cholesterol per mg magnetosome at 0.05 mg-mag/mL (Fig. 3B), corresponding to 7.9 wt.% of the total membrane lipid in 1 mg magnetosome. This enhancement may have resulted from improved particle dispersion and a higher relative abundance of the MβCD-Chol complex per particle. However, further decreasing the particle concentration may promote phospholipid extraction by excess MβCD, potentially destabilizing the membrane and reducing cholesterol retention. Collectively, these data show that *in vitro* cholesterol loading can be precisely tuned by adjusting the Chol:MβCD ratio and magnetosome concentration.

### Lipid remodeling enhances functional display of TrkA on magnetosomes

**Fig 4:**
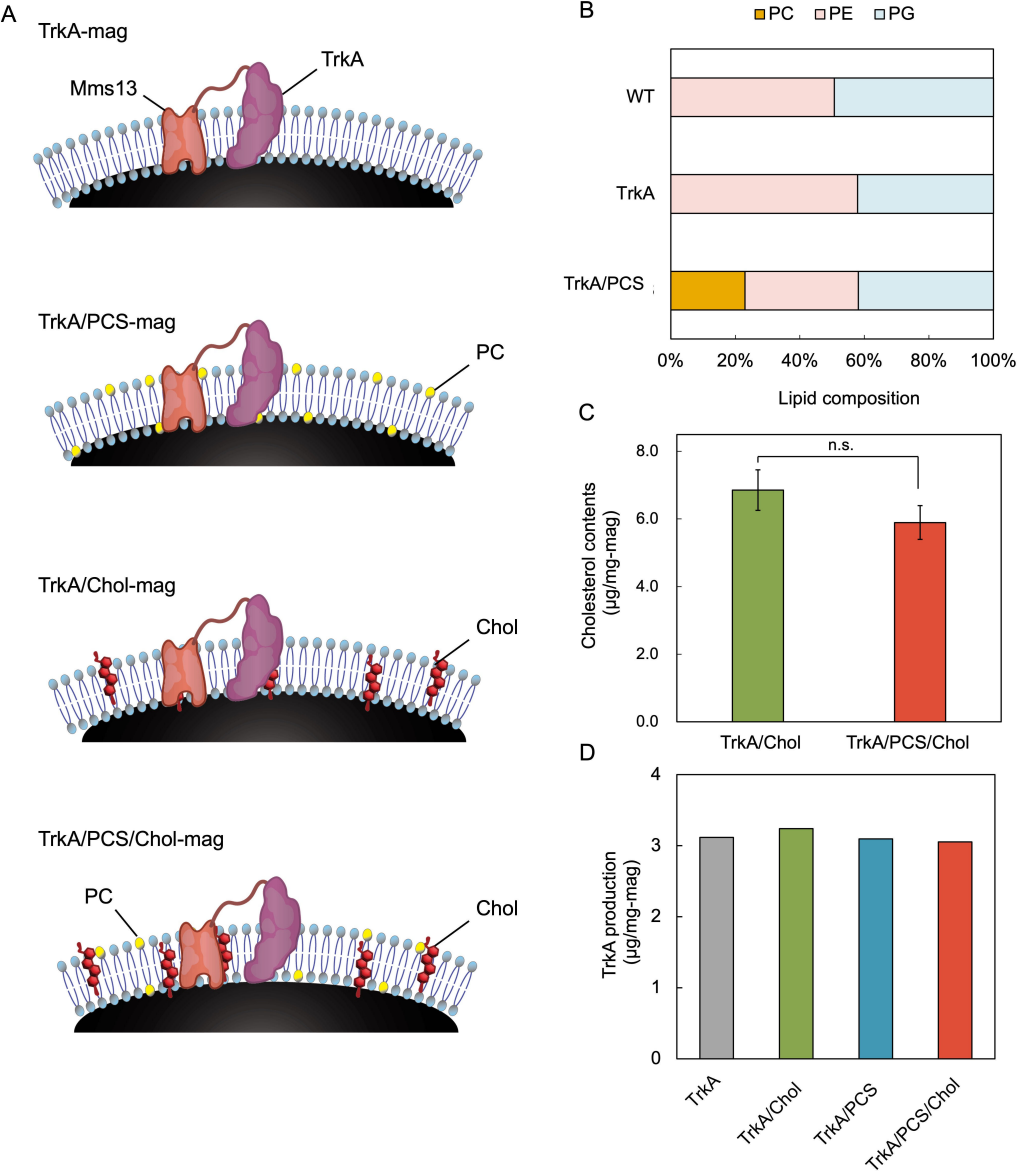
Membrane lipid remodeling of TrkA-displaying magnetosomes. (**A**) Schematic representation of the magnetosome membrane remodeled by dual-lipid incorporation. (**B**) Lipid composition of magnetosomes extracted from WT and transformants. Phosphatidylcholine (PC) was detected only in the TrkA/PCS strain, confirming successful *in vivo* incorporation. (**C**) Cholesterol loading into magnetosomes isolated from TrkA and TrkA/PCS strains (cholesterol:MβCD = 1.0:10 mM; 30 min of shaking; 0.05 mg magnetosomes/mL). Both strains incorporated comparable amounts of cholesterol. (**D**) Quantification of TrkA display levels. Band intensities were converted to absolute amounts using a calibration curve generated from FLAG-tagged standards using ImageJ software. TrkA abundance was unaffected by differences in the membrane lipid composition. Data are shown as mean ± SD (*n* = 3 biological replicates). Statistical significance was evaluated using a two-tailed Welch’s *t*-test; n.s., no significant difference.

Our previous study showed that introducing a plasmid encoding PCS into *M. magneticum* enables the *in vivo* incorporation of PC into the magnetosome membrane (15). To evaluate the effect of lipid remodeling on TrkA function, we combined *in vivo* PC biosynthesis with *in vitro* cholesterol loading (Fig. 4A). A tetracycline-inducible operon was constructed in which the Mms13-TrkA fusion and pcs were co-expressed, permitting simultaneous control of membrane protein display and lipid composition. TLC confirmed successful PC incorporation: magnetosomes from the TrkA/PCS transformant contained PC at 23.0 wt.% of the total lipids (Fig. 4B; Fig. S1). We then introduced cholesterol into magnetosomes from TrkA-expressing (TrkA-mag) and TrkA/PCS-expressing (TrkA/PCS-mag) strains. The resulting cholesterol content was 6.9 ± 0.6 µg/mg and 5.9 ± 0.5 µg/mg for magnetosomes, respectively (Fig. 4C). Western blotting confirmed TrkA expression in the magnetosomes of both strains. The band intensity corresponding to full-length Mms13-TrkA (~102 kDa) was comparable between the TrkA and TrkA/PCS transformants (Fig. 4D; Fig. S2), indicating that PC and cholesterol incorporation did not affect TrkA display efficiency. These results demonstrate that PC and cholesterol can be incorporated into magnetosome membranes without compromising the surface display of TrkA, enabling orthogonal control of the lipid environment and receptor presentation.

**Fig 5:**
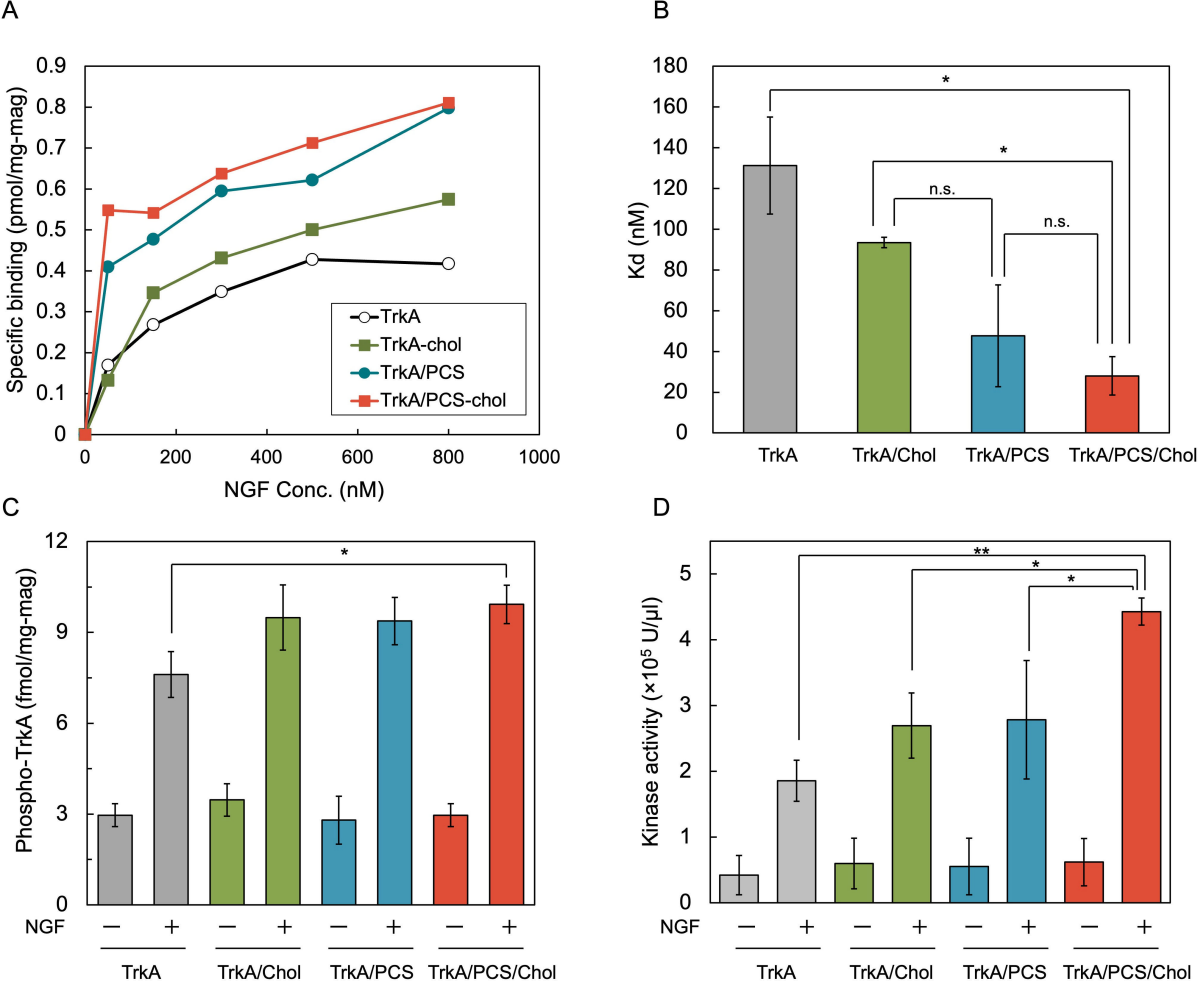
Functional analysis of TrkA on magnetosomes with a remodeled lipid composition. (**A**) NGF-binding assay. The luminescence signals obtained at each NGF concentration were normalized to those of the wild-type magnetosomes. PC and/or cholesterol incorporation increased NGF binding compared to that of the unmodified control. (**B**) Ligand affinity expressed as the dissociation constant (Kd). Lower Kd values indicate stronger NGF binding. TrkA displayed on magnetosomes containing both PC and cholesterol exhibited the highest affinity. (**C**) Autophosphorylated TrkA levels were detected using anti-TrkA (phospho-Y490) IgG. (**D**) TrkA kinase activity was measured after NGF stimulation, followed by ATP addition and detection with anti-phosphotyrosine (PY20)-HRP. The dual incorporation of PC and cholesterol resulted in the greatest increase in kinase activity. Data are shown as mean ± SD (*n* = 3 biological replicates). Statistical significance was evaluated using a two-tailed Welch’s *t*-test; *P < 0.05; **P < 0.01; n.s., no significant difference.

Numerous studies have highlighted the importance of the NGF/TrkA axis in cancer progression (27, 28). Next, we evaluated the impact of membrane remodeling with PC and cholesterol on TrkA-ligand interactions. As shown in Fig. 5A, TrkA displayed on PC-enriched magnetosomes exhibited higher specific binding to NGF than that displayed on unmodified membranes. This enhancement mirrors our previous findings on TSHR (15). Cholesterol incorporation alone also improved NGF binding, and the combination of PC and cholesterol resulted in the highest specific binding. Scatchard analysis revealed a Kd of 130 nM for TrkA on unmodified magnetosomes, which improved to 47 nM with PC (TrkA/PCS), 93 nM with cholesterol alone (TrkA/Chol), and 28 nM with both PC and cholesterol (TrkA/PCS/Chol-mag) (Fig. 5B). As lower Kd values indicate a higher binding affinity, these results demonstrate a 4.7-fold improvement in ligand affinity through dual-lipid incorporation. In the early kinase-focused hit discovery phase of a lead identification program, potency benchmarks typically target ≤100 nM (29); therefore, the TrkA Kd of 28 nM on PC/Chol-modified magnetosomes meets the practical threshold for primary hit screening. Similar to other receptor tyrosine kinases, TrkA-ligand interactions trigger dimerization, autophosphorylation, and downstream signaling activation (30). Aberrant TrkA activity contributes to oncogenesis, and TrkA phosphorylation inhibitors are currently under development (31). Therefore, we assessed TrkA autophosphorylation. A modest 1.5-fold increase in phosphorylation was observed in TrkA/PCS/Chol compared to TrkA alone, whereas TrkA/Chol and TrkA/PCS showed similar levels to the unmodified control (Fig. 5C). These findings indicate that the co-incorporation of PC and cholesterol into magnetosome membranes enhances both ligand binding and the signaling capacity of magnetosome-displayed TrkA.

As TrkA is a receptor tyrosine kinase, several inhibitors targeting TrkA activity, particularly in the context of TrkA gene fusions or overexpression in cancer, have been developed (32). Therefore, we assessed TrkA tyrosine kinase activity in our system. Following NGF stimulation, the kinase activity of TrkA displayed on PC- and cholesterol-modified magnetosomes was the highest among all conditions, showing a 2.7-fold increase compared to that of native TrkA magnetosomes (Fig. 5D). Incorporation of PC or cholesterol alone yielded only modest improvements, but their combination resulted in synergistic enhancement. Although the underlying mechanism remains unclear, two models have been proposed to explain the cholesterol-mediated regulation of membrane proteins. The first is an indirect mechanism, in which cholesterol alters the physical properties of the membrane, such as fluidity, lateral pressure, and lipid packing (33). These changes can stabilize or destabilize the conformation of transmembrane receptors. Cholesterol- and sphingolipid-enriched microdomains, such as lipid rafts and caveolae, spatially confine receptors and influence their function; however, their existence on bacterial-derived magnetosome membranes has not been confirmed.

The second is a direct mechanism in which cholesterol interacts with specific amino acid motifs within the receptor. The cholesterol recognition/interaction amino acid consensus (CRAC) motif is one such feature, and direct binding between cholesterol and CRAC residues has been proposed to modulate receptor structure and activity (34, 35). Cholesterol can also weakly associate with PC in the membrane, allowing its hydroxyl group to directly access the extracellular domains of membrane proteins (36). Thus, it is plausible that in PC- and cholesterol-modified magnetosomes, cholesterol directly interacts with TrkA, contributing to structural changes and enhanced function. TrkB, a close homolog of TrkA, is also regulated by membrane dynamics. A recent study reported that antidepressants bind to the transmembrane domain of TrkB and that a mutation at residue S441 alters receptor conformation and activity (37). However, despite possessing a CRAC motif, TrkB does not exhibit significant cholesterol-dependent conformational changes, suggesting that cholesterol sensitivity is both receptor- and context-dependent. Further studies, such as NMR spectroscopy, are necessary to determine whether cholesterol directly interacts with TrkA and induces conformational changes that underlie the observed functional enhancement.

TRK gene fusions are estimated to occur in approximately 17%–20% of all cancers (38). Aberrant TRK signaling has been identified as a key driver of oncogenesis in TRK fusion-positive tumors, and small-molecule inhibitors targeting TRK kinase domains have emerged as promising therapeutic strategies. To enable rational drug discovery targeting TRK pathways, it is essential to obtain functionally active TrkA for binding and functional assays. However, producing membrane-bound receptor tyrosine kinases at scale is challenging because of their complex transmembrane architecture. Furthermore, membrane disruption during purification can compromise receptor integrity and alter its native function (39). Mounting evidence suggests that lipid composition strongly influences receptor–ligand interactions and downstream signaling responses (40–42). These findings underscore the need to consider the membrane context in future drug screening platforms.

### Conclusions

In this study, we demonstrated that the magnetosome display system allows for the efficient presentation of TrkA on the surface of magnetic nanoparticles. By incorporating phosphatidylcholine and cholesterol, we engineered the magnetosome membrane to mimic the lipid environment of human cell membranes more closely. These modifications significantly improved the binding affinity, phosphorylation efficiency, and kinase activity of TrkA. This integrated strategy expands the metabolic and synthetic biology toolkit for producing functional membrane proteins on scalable magnetosomes and establishes a cost-effective platform for drug discovery efforts targeting TrkA and other membrane receptors.

## Data Availability

Data will be made available on request.

## SUPPLEMENTAL MATERIAL

Figures S1 and S2 aem.01710-25-s0001.docx Phospholipid and western blot analysis.

ASM does not own the copyrights to Supplemental Material that may be linked to, or accessed through, an article. The authors have granted ASM a non-exclusive, world-wide license to publish the Supplemental Material files. Please contact the corresponding author directly for reuse.
