# Supplementary material for: Orthogonal in vivo and in vitro membrane engineering enables human-like lipid remodeling of bacterial magnetosomes for functional TrkA display
Source: Appl Environ Microbiol. 2025 Nov 5;91(12):e01710-25. doi: 10.1128/aem.01710-25 (PMC12724377; doi:10.1128/aem.01710-25)
Supplement: Figures S1 and S2 — Phospholipid and western blot analysis. [file aem.01710-25-s0001.docx]

Supplemental information

Orthogonal *in vivo* and *in vitro* membrane engineering enable human-like lipid remodeling of bacterial magnetosomes for functional TrkA display

Ryoto Tomoe, Shunya Waki, Keita Morimoto, Takaho Ogaki, Tsuyoshi Tanaka, and Tomoko Yoshino#

Division of Biotechnology and Life Science, Institute of Engineering, Tokyo University of Agriculture and Technology, Koganei, Tokyo, Japan

Running Head: Orthogonal Lipid-Remodeled Magnetosomes for TrkA Display

#Address correspondence to Tomoko Yoshino: [y-tomoko@cc.tuat.ac.jp](mailto:y-tomoko@cc.tuat.ac.jp)

Fax: +81-42-385-7713

Phone: +81-42-388-7021


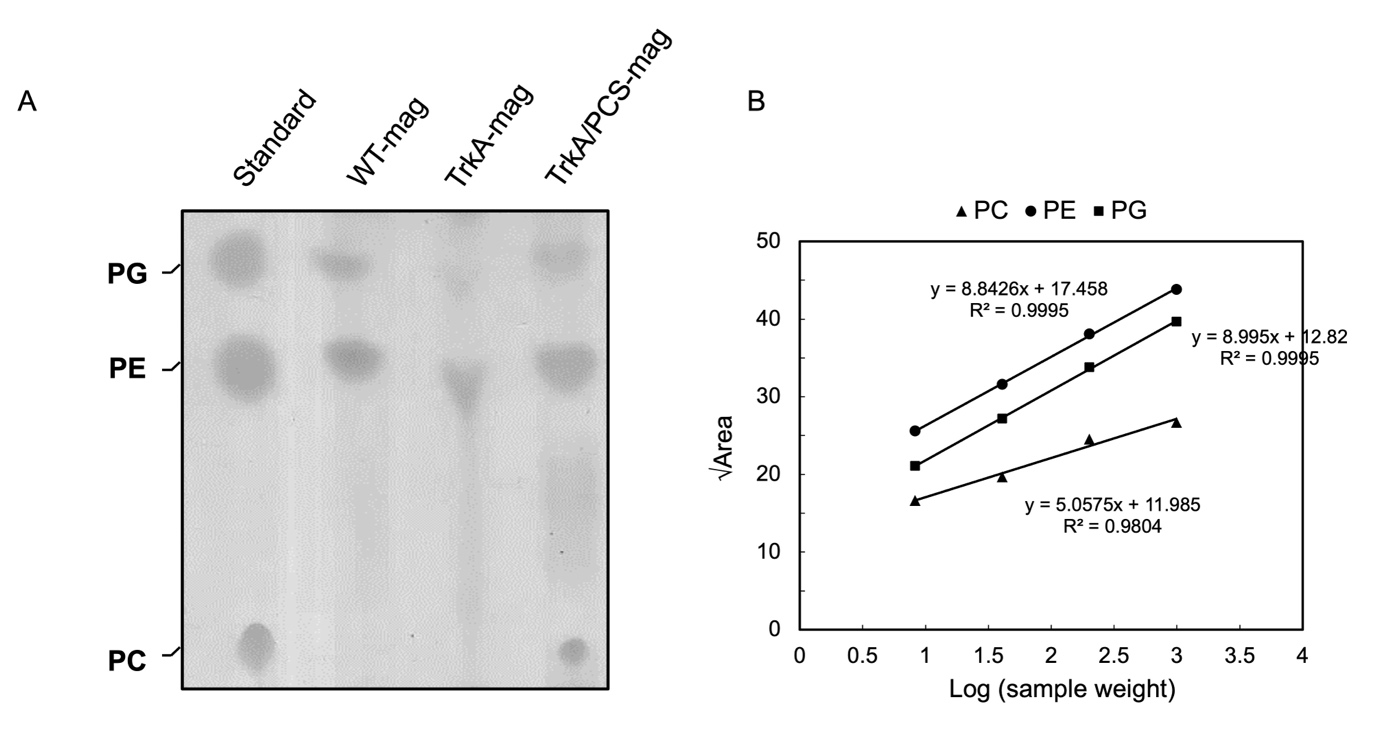
 **Fig. S1.** Analysis of the phospholipid composition (weight fraction) of the magnetosome membranes. **(A)** Thin-layer chromatography (TLC) image of lipids extracted from magnetosomes. WT-mag and TrkA-mag mainly comprised phosphatidylethanolamine (PE) and phosphatidylglycerol (PG), whereas TrkA/PCS-mag comprised PE, PG, and phosphatidylcholine (PC). **(B)** Standard curve used for lipid quantification. Known amounts of PC, PE, and PG were spotted onto the TLC plate; spot areas were measured, and the resulting calibration was applied to determine the mass of each lipid species in the unknown samples.


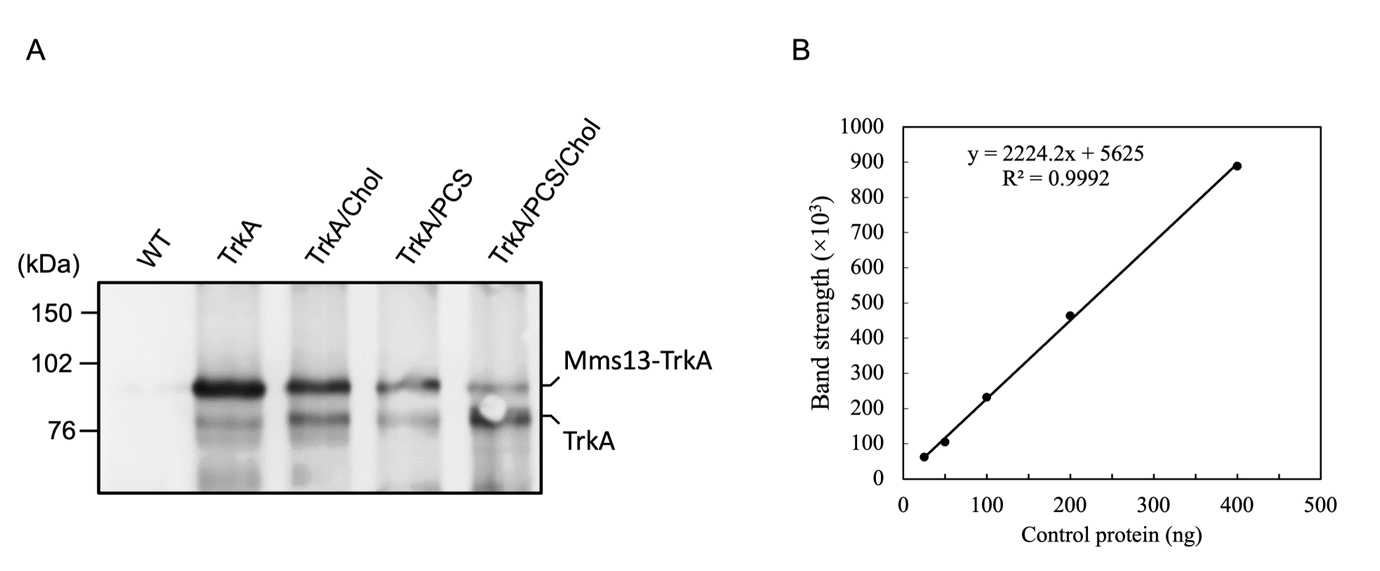
 **Fig. S2.** Western blot analysis and quantification of TrkA displayed on magnetosomes. **(A)** Western blot analysis of TrkA on magnetosome membrane. An anti-FLAG antibody targeting the C-terminal tag detected the full-length Mms13-TrkA fusion protein (~100 kDa). The smaller band observed in both samples likely represents cleaved TrkA due to partial dissociation from Mms13 during protein extraction. **(B)** Standard curve generated from known amounts of FLAG-tagged control protein and used to quantify TrkA. Band intensities for each transformant were background-corrected by subtracting the WT signal and then converted to absolute amounts using a standard curve.
